# On the dynamic and even reversible nature of Leigh syndrome: Lessons from human imaging and mouse models

**DOI:** 10.1016/j.conb.2021.09.006

**Published:** 2021-10-14

**Authors:** Melissa A. Walker, Maria Miranda, Amanda Allred, Vamsi K. Mootha

**Affiliations:** 1 Howard Hughes Medical Institute, Department of Molecular Biology, Massachusetts General Hospital, United States; 2 Broad Institute of Harvard, MIT, United States; 3 Department of Neurology, Massachusetts General Hospital, United States

## Abstract

Leigh syndrome (LS) is a neurodegenerative disease characterized by bilaterally symmetric brainstem or basal ganglia lesions. More than 80 genes, largely impacting mitochondrial energy metabolism, can underlie LS, and no approved medicines exist. Described 70 years ago, LS was initially diagnosed by the characteristic, necrotic lesions on autopsy. It has been broadly assumed that antemortem neuroimaging abnormalities in these regions correspond to end-stage histopathology. However, clinical observations and animal studies suggest that neuroimaging findings may represent an intermediate state, that is more dynamic than previously appreciated, and even reversible. We review this literature, discuss related conditions that are treatable, and present two new LS cases with radiographic improvement. We review studies in which hypoxia reverses advanced LS in a mouse model. The fluctuating and potentially reversible nature of radiographic LS lesions will be important in clinical trial design. Better understanding of this plasticity could lead to new therapies.

## Introduction

Leigh syndrome (LS), also known as subacute necrotizing encephalomyelopathy, is the most common pediatric manifestation of inherited mitochondrial disorders [1]. In 1951, Dennis Leigh described a 5-month old male infant with subacute onset of progressive somnolence. He died at age 7 months, afebrile but diaphoretic with unreactive pupils, marked hypertonia, with upgoing toes but absent deep tendon reflexes. Autopsy revealed bilateral, symmetric brainstem, basal ganglia, and spinal cord gray matter lesions characterized by gliosis, vacuolation, capillary proliferation, and relative sparing of neurons within areas with severe necrosis [2].

As more cases of LS began to be recognized, there was growing appreciation that this is a metabolic disease. Acidosis, as indicated by low serum bicarbonate in patients in the 1950s and subsequently lactic acidosis found in patients in the 1960s, was the first salient biochemical observation [3]. Defects in pyruvate metabolism were subsequently documented in several patients with LS. Although defects in pyruvate metabolism were initially thought to be related to pyruvate carboxylase deficiency, this hypothesis was never proven, and many of these cases were ultimately linked to pyruvate dehydrogenase deficiency [4]. Cytochrome *c* oxidase deficiency became the first component of the electron transport chain (ETC) to be linked to LS in 1977 [5].

Our diagnostic approach and molecular genetic understanding of LS have dramatically improved over the past few decades. LS was diagnosed based on autopsy findings till the 1970s when the advent of brain contrast tomography (CT) and, subsequently, magnetic resonance imaging (MRI) enabled antemortem diagnosis [6–9]. With advances in biochemical and molecular genetic testing, scores of mitochondrial biochemical and genetic lesions were linked to LS. Today, LS is widely recognized as the most common pediatric manifestation of mitochondrial disease, with greater than 80 genes — mostly encoding proteins localized to mitochondria — underlying this syndrome [1].

Lake et al. have most recently defined LS as i) a characteristic clinical presentation including psychomotor retardation and/or regression with progressive neurologic decline, often in a stepwise fashion, with decompensations, frequently with illness, ii) radiologic evidence of LS lesions in the basal ganglia or brainstem nuclei, which appear hyperintense in T2-weighted MRI sequences, iii) biochemical evidence of abnormal energy metabolism, and iv) identification of a pathogenic variant in a characteristic gene [1]. ‘Leigh-like’ disease or “LS spectrum” is used to describe cases in which a subset of these features including the radiographic or histologic findings are fulfilled [1]. At present, the precise sequence of events linking mutations in disease genes to end brain disease is not known. A better understanding of this molecular pathogenesis is crucial as, at present, there are no proven therapies.

Here, we review the published literature, present two new clinical cases, and integrate recent animal studies that collectively suggest that the brain disease in LS may be more plastic than previously appreciated. In particular, we explore the question of whether the antemortem MRI abnormalities may not fully equate to end-stage pathology and that these radiographic findings might instead reflect intermediate — potentially reversible — stages of this disease.

### Radiographic brain lesions in Leigh syndrome

Hyperintense signals on the T2-weighted MRI sequence can arise from multiple processes, making the origin of LS lesion signal abnormalities unclear. Tissues such as cerebrospinal fluid (CSF) that typically have high water content appear bright or white on T2-weighted sequences, whereas brain matter appears in shades of gray varying by composition. LS lesions are T2 hyperintense, as they represent an uncharacteristically bright signal in affected regions [10]. What processes typically produce T2 hyperintensity? Possibilities include hemorrhage, ischemia, neoplasm, infection, and inflammation [10]. Reduced diffusivity on isotropic diffusion mapping (thought to represent cytotoxic edema) [11] has occasionally been reported in LS lesions (e.g. the study reported by Bonfante et al. [9]). Notably, however, not all studies included this technique; and this abnormality can also represent an artifact of high T2 signal. While the contribution of hemorrhage, neoplasm, or infection to the T2 signal abnormalities in LS can be definitively excluded by autopsy and clinical studies, the relative contributions of inflammation, edema, loss of plasma membrane integrity, and/or frank necrosis to high T2 signals remain unknown in both the end-stage disease and antemortem intermediate states in humans.

Although LS lesions are quite distinctive, a small handful of hereditary conditions and a number of acquired conditions — ranging from toxins, insults, nutritional deficiencies, infections — can lead to lesions with MRI features resembling LS (Table 1) [1,12–35,54,56]. These mimetics are not only important considerations in the differential diagnosis, but also promise to provide insight into the etiopathogenesis of LS.

The rare LS cases reporting both neuroimaging and subsequent autopsy findings are instructive and reveal histologic evidence of edema and capillary proliferation, gliosis, and — to a lesser extent — inflammation. Kissel et al. [36] reported an apparent adult-onset LS case with progressive T2 prolongation signal changes of the cerebral peduncles, periaqueductal gray, and basal ganglia with initial expansion, followed by contraction of the latter. Microscopic evaluation on autopsy reviewed capillary proliferation and gliosis in regions with relative neuronal preservation, as well as lipid-laden macrophages in areas of complete neuronal replacement by cavitation and necrosis. All areas affected on histopathologic examination were also detected on MRI [36]. Koch et al. [7] reported a case of twin infants with LS where CT scan lesions observed in late stages also correlated with loss of cellularity, vascular engorgement, and gliosis on autopsy.

### Resolution of radiographic brain lesions in patients with Leigh syndrome

Serial imaging has at times demonstrated regression of some radiographic LS lesions. Indeed, as early as 1985, Koch et al. reported the aforementioned case of twins diagnosed with LS on head CT. In that study, some of the lesions appeared to regress on repeat imaging. Initial CT demonstrated hypodensities of the bilateral basal ganglia and midbrain tegmentum at age 10 months. The basal ganglia lesions were not, however, apparent on repeat imaging at 17 months despite persistent decline in respiratory function. Autopsy performed on one twin showed that the basal ganglia lesions that had resolved on repeat imaging demonstrated no gross necrosis, only mild neuronal loss, capillary proliferation, and reactive astrocytosis. However, in the brainstem, where imaging abnormalities had been persistent, there was ‘significant’ loss of cells, vascular ‘engorgement,’ and ‘marked’ reactive astrocytosis [7]. These findings led the authors to hypothesize that ‘active lesions with vascular proliferation’ but without frank necrosis might appear and subsequently resolve [7,8]. Intriguingly, recent reports of arterial spin labeling in patients with LS indicate increased blood flow to LS regions during acute symptomatic crises [37]. Others have similarly observed radiologic improvement of T2 signal abnormalities without evidence of necrosis in various cohorts [9,38,39] and in case report format (Table 2). These findings suggest that LS lesions that regress on repeat imaging potentially correspond to intermediate pathologic states rather than end-stage necrotic lesions.

**Figure 1:**
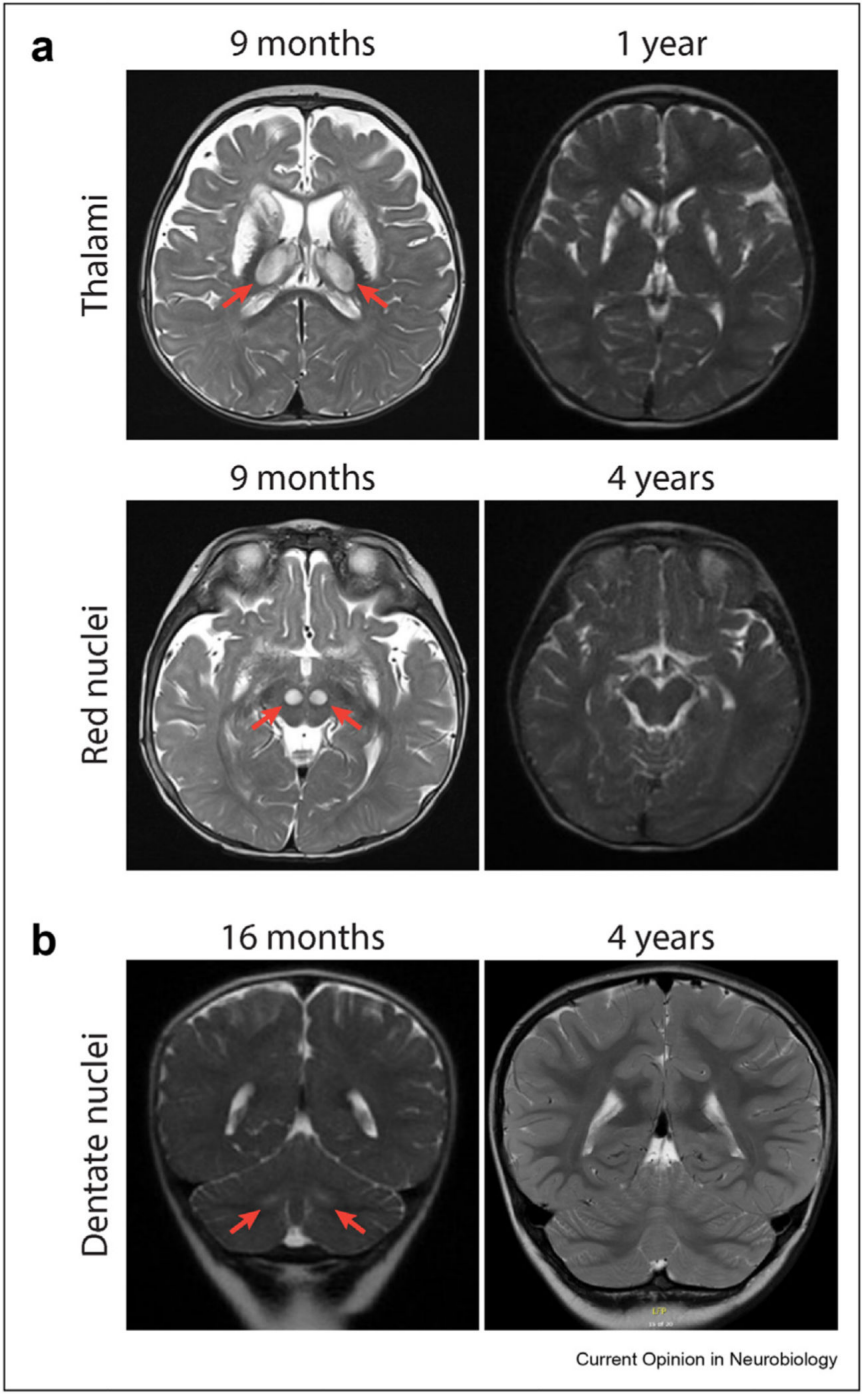
T2-weighted MRI showing resolution of lesions. (**a**) Patient 1 basal ganglia and thalamic lesions at age 9 months with resolution of thalamic lesions at age 4 years (top) and red nuclei lesions at 9 months with resolution at age 4 years (bottom). (**b**) Patient 2 demonstrates improvement of dentate nuclei hyperintensities at age 16 months with marked improvement at age 4 years. MRI, magnetic resonance imaging.

We report two new cases of transient LS brain lesions. **Case 1** is a 10-year-old girl who first presented at age 9 months with failure to thrive and stridor. The MRI brain obtained at that time revealed T2 hyperintense bilateral lesions of the caudate nuclei and putamina, thalami, and red nuclei. CSF lactate was elevated at ∼5 mM. DNA sequencing revealed compound heterozygous mutations in mitochondrial complex I (CI) assembly factor *NDUFAF3* (c.489_490delTG and p.Y11D). She began taking α-Tocotrienol quinone in a clinical trial as a toddler and continues on this medication. At age 4 years, she was nonambulatory but had made significant developmental progress, with 2–3 spoken words and the ability to point to multiple people or body parts on command. The T2 half-Fourier single-shot turbo spin-echo (HASTE) brain sequence demonstrated the decreased total area of T2 hyperintensity of the basal ganglia lesions and apparent regression of basal ganglia, thalami, and red nuclei signal abnormalities (Figure 1A). **Case 2** is a 9-year-old boy who initially presented at age 16 months with ataxia, decreased arousal, and seizures. The MRI brain at that time demonstrated bilateral, symmetric T2 hyperintensities of the dentate nuclei, and lactate peaks on magnetic resonance spectroscopy. Enzyme and genetic testing confirmed defects in the pyruvate dehydrogenase complex subunit *PDHA1* (hemizygous p.L5P), and ketogenic diet was initiated. The repeat MRI brain at age 4 years showed interval decrease in T2 hyperintense lesions (Figure 1B). At age 8 years, he speaks in full sentences and ambulates independently.

We identified more than 32 published cases of LS with radiographic resolution of some or all lesions [7–9,38–50,52–54] (Table 2). Age of onset ranged from birth to 22 years. Twenty-one patients carried a genetically confirmed diagnosis. Eight patients carried mitochondrial DNA (mtDNA) mutations, 13 had nuclear gene defects, with only two of these occurring in genes functioning outside of the mitochondrion (*SLC19A3* [54,55] and *TPK1* [56]). Transient lesions were located either in the basal ganglia (18/30), the brainstem (1/30), or in multiple brain regions (basal ganglia and the brainstem [4/30], basal ganglia and thalami [1/30], basal ganglia and the cerebellum [4/30], the brainstem and thalami [2/30]). Although most cases describe bilateral regression of lesions, asymmetric resolution of caudate lesions was observed in two patients. Transient lesions often recurred in the setting of illness or metabolic crises, which are well known to be preciptants of disease progression [40,41,48]. Among published cases, 16 patients’ improved MRIs coincided with clinical improvement at the time of publication [8,38,40,42–46,48,49,52–54]. In some cases, this improvement was coincident with a treatment including ketogenic diet [38,44], rapamycin/steroids/n-acetylcysteine [52], coenzyme Q10 [49], or biotin and thiamine supplementation [54,57]. Critically, no contemporaneous histology has been reported for these intermediate states in which T2 hyperintensity had been observed but had later regressed, and the availability of associated clinical data is variable making it difficult to correlate imaging findings with clinical course.

### Inherited or acquired thiamine deficiency: a special case of treatable lesions in humans

Owing to shared features in the disease mechanism (Table 1), clinical presentation, radiographic, and histological findings, comparison of LS to Wernicke Korsakoff syndrome (WKS) — which is often reversible with treatment and comparatively well-understood — merits special attention. WKS results from dietary deficiency of and is treated with supplementation of the essential vitamin thiamine (B1), which is a coenzyme component for several mitochondrial enzymes, including the pyruvate dehydrogenase complex, a known genetic cause of LS [1]. It is increasingly recognized that WKS has two phenotypes: one arising purely from nutritional deficiency; and a nutritional deficiency in the setting of alcohol use disorder [31]. The former presents in the acute phase (Wernicke encephalopathy) with hearing loss in addition to the classic triad of confusion, oculomotor dysfunction, and ataxia, although hearing impairment is not a feature of alcohol use disorder-related disease [58]. In WKS, T2 hyperintensities are found in the bilateral mammillary bodies, thalami, tectal plate, and periaqueductal gray matter. Notably, nonalcohol use disorder patients — who are more frequently pediatric — may additionally have signal abnormalities of the basal ganglia, particularly the putamina [31]. The mammillary bodies are uniformly affected in WKS but never to our knowledge in LS (Table 1). The radiographic appearance and postmortem histology of WKS and LS are similar, with both characterized by relative neuronal sparing and striking gliosis, but unlike LS, WKS often also demonstrates evidence of prior hemorrhage [59]. With prompt thiamine supplementation [31], most patients with WKS will experience significant clinical recovery, and resolution of brain lesions after treatment has been reported [60].

Intriguingly, although most LS disease genes encode mitochondrial proteins involved in energy metabolism, two of the genetic causes of LS involve perturbations of thiamine metabolism (Table 1), raising the possibility of shared pathogenesis between WKS and LS. LS-linked genes *SLC19A3* and *TPK1* encode the plasma membrane and cytosolic proteins required for thiamine transport into cells and its processing, respectively. In contrast to WKS, mutations in these genes lead to lesions in brain regions entirely consistent with LS and comprise the very small subset of treatable forms of LS that exhibit reversibility with high doses of thiamine [56,61].

### Improvement of Leigh syndrome brain disease in a mouse model

The best-characterized animal model of LS is the mitochondrial CI accessory subunit *Ndufs4* knockout (*Ndufs4*^−/−^) mouse developed by the Palmiter laboratory [62]. CI deficiency is a common cause of LS, and mutations in *NDUFS4* can, rarely, cause LS in humans [63]. *Ndufs4*^−/−^ mice are born at term and show little or no apparent disease till about 5 weeks when they develop progressive encephalopathy and die around 7 weeks [64]. The *Ndufs4*^−/−^ have reduced body weight and temperature, ataxia, seizures, lethargy, blindness, and irregular breathing [64,64,65]. These mice typically develop bilateral symmetric gray matter lesions in the brainstem (vestibular nuclei), cerebellum, and olfactory bulb. Although the neuroanatomy of these lesions is consistent with the aforementioned definition of LS [1], vestibular nuclei lesions have also specifically been reported in humans [68], and cerebellar lesions are a common feature in patients with LS, it is notable that these mice do not exhibit lesions in the basal ganglia [64]. Similar to humans, the *Ndufs4*^−/−^ lesions are characterized by gliosis with prominent microglial activation, vascular proliferation, neuronal loss, and vacuolation [64]. Furthermore, the lesions identified by histopathology correlate with T2-weighted MRI hyperintensities [65]. Other murine mitochondrial encephalopathy models have been reported [69–72], but the correlation of the histopathology with radiologic findings has only been firmly established for the *Ndufs4*^−/−^.

Multiple therapeutic approaches have been tested in the *Ndufs4*^−/−^ mouse. Recent gene therapy strategies to re-express mouse *Ndufs4* systemically restored lifespan and healthspan in the *Ndufs4*^−/−^ [73]. Repletion of nicotinamide adenine dinucleotide (NAD+) provided moderate extension of the the *Ndufs4*^−/−^ lifespan from 60 to 100 days [74,75]. Interestingly, ectopic expression of the yeast NADH dehydrogenase NDI1 in the brain rescued the lifespan of the brain-specific *Ndufs4* knockout, but the mice still had severe ataxia [76]. It is unclear whether the beneficial effect of NDI1 is specific to restoring NAD+ redox balance or of other downstream effects of electron transport chain inhibition (i.e. redox of the coenzyme Q pool, oxygen consumption, proton pumping by downstream complexes, or mitochondrial ATP). Surprisingly, targeting reactive oxygen species with a potent antioxidant (KH176) [77] or transgenic expression of metallothionein 1 [78] had no effect on lifespan. Rapamycin and doxycycline improved healthspan and doubled the lifespan of *Ndufs4*^−/−^ [79–82].

The most powerful intervention to date in this model, comparable to gene therapy, has been chronic, continuous exposure to mild hypoxia (11% oxygen), which led to a dramatic extension of lifespan and healthspan, with these mice now living for more than 270 days. Moreover, radiologic (T2 MRI signal abnormalities) and histologic staining of neuroinflammatory microglial marker ionized calcium binder adaptor moleculare-1 (IBA1) show no evidence of the lesions in hypoxia-treated mice [83,84]. Although rapamycin, doxycycline, and hypoxia prevent neuroinflammation, anti-inflammatory drug tacrolimus had no beneficial effect [79] suggesting that the mechanism(s) of action of successful interventions go beyond blocking inflammation. To our knowledge, spontaneous recovery or reversal — partial or complete, by imaging or histology — has never been documented in this mouse model.

**Figure 2:**
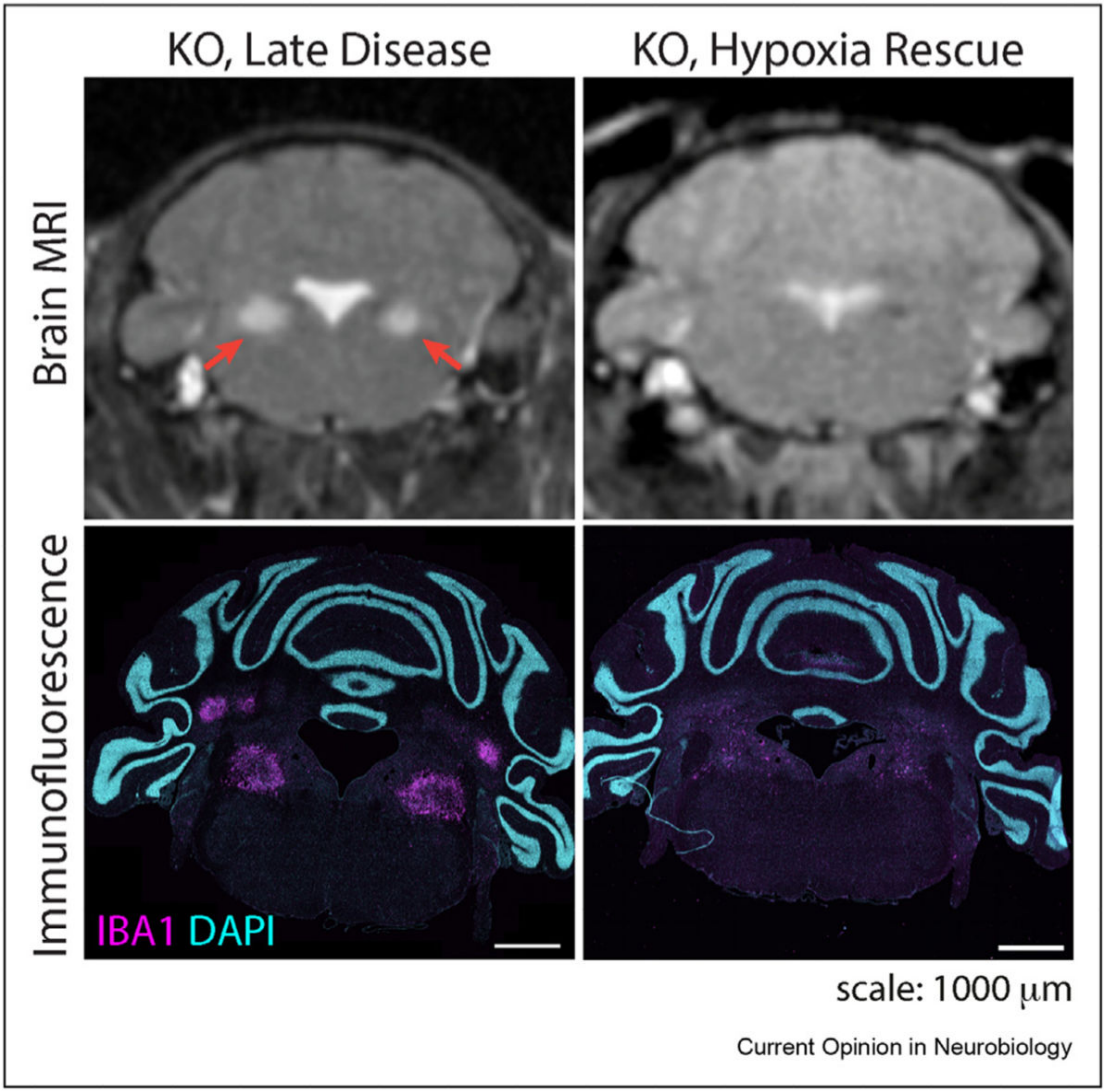
Radiologic and histopathologic reversal of lesions in the *Ndufs4*^−/−^ mouse after hypoxia treatment. Top: T2 MRI; red arrows indicate hyperintensities in vestibular nuclei. Bottom: immunohistochemistry of coronal section labeling microglia with IBA1 and the nuclear counterstain DAPI (4′,6-diamidino-2-phenylindole). MRI adapted from Ferrari et al., 2017.

Treatment of *Ndufs4*^−/−^ with hypoxia (11% oxygen) initiated at advanced disease (7 weeks), when the mice present advanced symptoms including radiologic abnormalities and are close to fulfilling humane euthanasia criteria, clinically reverses disease and rescues the mice by improving their overall body weight and motor function, as well as substantially extending their lifespan. Given the uniformly progressive nature of the disease in this mouse model and that neurodegeneration has been reported at this timepoint [64], this dramatic improvement was surprising. Remarkably, in addition to clinical improvement, T2 MRI hyperintensities progressively decrease till they become almost undetectable after one month of hypoxia treatment, which correlates to a substantial reduction of neuroinflammation in histopathology at this age [84] (Figure 2).

Although the mechanism of LS lesion improvement in the *Ndufs4*^−/−^ remains unknown, partial reversibility in WKS disease rodent models has been reported. In a thiamine-deficient mouse model, thiamine treatment prevented further neuroinflammation and neuronal death only when administered before extensive neuronal loss occurs [85], indicating that treatment halts disease progression but does not fully reverse it, suggesting that inflammation preceding necrosis may be amenable to intervention. Differences in the ‘lesion age’ may explain why thiamine treatment in the analogous rat model normalized radiologic hyperintensities in the thalamus and colliculi but not in mammillary nuclei and lateral ventricles [86]. Unlike thiamine rescue in WKS models, pathologic phenotype, imaging abnormalities, and histology are all ameliorated by hypoxia even when it is implemented at very advanced disease stages and does not restore the primary CI biochemical defect in the *Ndufs4*^−/−^ [83].

Radiographic improvement in lesions in the *Ndufs4*^−/−^ has also been achieved experimentally with other interventions that reduce brain oxygen delivery including anemia and administration of sublethal carbon monoxide but not with constitutive activation of the hypoxia-inducible factor (HIF) pathway [87]. *Ndufs4*^−/−^ mice exhibit a high partial pressure of brain oxygen, and hyperoxia worsens disease, potentially reflecting reduced oxygen extraction by brain mitochondria [87], a finding reminiscent of the decreased oxygen utilization observed in patients with mitochondrial myopathies [88].

### Future outlook

In the 70 years since the initial description of LS, the field has made tremendous progress both in antemortem diagnosis enabled by MRI imaging and in defining more than 80 genetic causes for this disease. Although we typically think about LS as being a uniformly progressive and lethal disease, as we have discussed, there are a growing number of clinical case reports and now mouse models that support the notion that some of the antemortem imaging abnormalities observed in LS may not represent end-stage disease but may in fact represent an intermediate state in the disease that is far more dynamic and even reversible. In particular, mouse studies suggest that neuroinflammation is one of the features that is reversible and correlates with phenotypic and radiographic improvement.

At present, we do not have a clear understanding of how inherited mutations in any of greater than 80 different genes can lead to radiographically supported LS. Key open questions are as follows: (1) which are the dynamic neuropathological features of LS lesions evidenced by T2 MRI (e.g. neuroinflammation, edema)? (2) in addition to neuroinflammation, which aspects of intermediate disease are reversible? (3) why do infections or illness precipitate these lesions on imaging? (4) is there a critical treatment time window before irreversible tissue damage sets in? (5) is improvement caused by removal of pathogenic drivers (e.g. excess oxygen), by halting neuropathological effects of mitochondrial dysfunction (e.g. neuroinflammation), or instead by activating brain repair pathways?

The answers to these important questions remain unknown, but a detailed study of the human and mouse model literature yields a handful of common themes. As expected, repletion of cofactor deficiencies such as thiamine correlates temporally with brain lesion improvement in humans and rodents. It has also been shown that interventions to either buffer consequences of oxidative phosphorylation defects (e.g. redox imbalances) or reduce energetic demands (e.g. via mechanistic target of rapamycin (mTOR) inhibition) can delay brain disease and extend life in the mouse. Finally, capillary proliferation on histopathology and hyperperfusion of lesions during crises in humans supports a role for brain microvasculature in LS irrespective of genetic etiology. Strikingly, the radiographic differential diagnosis for LS lesions (Table 1) includes multiple processes in which pathogenic vasogenic edema — which may be associated with inflammation — has been implicated. These findings are of course insufficient to determine if hyperperfusion or any of its resultant effects — notable excess oxygenation — is causal or merely secondary findings in LS. However, bench research has produced exciting evidence that the presence of local hyperoxia — presumably unused oxygen substrate in the setting of deficient oxidative phosphorylation — may be driving disease progression and that removing it by various mechanisms improves disease phenotypes, neuroimaging, and even histology after disease onset. Having mouse models that share biochemical, histopathological, and radiographic features of LS combined with interventions that can tune the dynamics of the lesion will be a valuable resource to start addressing the key questions to understand and hopefully harness the dynamic nature of LS lesions for treatment.

Finally, it is critical to appreciate the dynamic nature of LS brain lesions in the context of therapeutic discovery and approval. If the underlying biology of the intermediate states of disease and their reversibility can be understood, it could imply that we can identify risk factors to be avoided and hopefully new medicines that directly target this biology for treating the scores of patients. Although such interventions may not fix the proximal genetic lesion, they could be very powerful in preventing disease progression. Mitochondrial diseases are extremely rare and heterogeneous, and although interventions in the past have been coincident with recovery in LS, we emphasize that most of the cases of radiographic improvement that we have reviewed here (Table 2) occurred spontaneously (e.g. studies reported by Koch et al. [7], Koch et al. [8], Arii and Tanabe [39], Sofou et al. [41], Roig et al. [43], and Alves et al. [50]). This underscores the need for proper natural history studies and rigorous control arms in clinical trials [89].

### Patient cases

Written consent to publish case information was obtained from patients.

## Figures and Tables

**Table 1:** Radiographic differential diagnosis of Leigh syndrome brain lesions.

**Leigh and Leigh-like syndrome**		
Mutations in >80 disease genes related to mitochondrial energy metabolism *(e.g.,*	T2 hyperintense bilaterally symmetric brainstem nuclei or basal ganglia lesions; lesions are frequently also found in the cerebellum and spinal cord.	[1]
PDH, OXPHOS)		
Genetic defects in thiamine transport/ processing *(SLC19A3* and *TPK1)*	T2 hyperintensities of bilateral basal ganglia, deep gray nuclei; *SLC19A3-* related disease also involves cortical and subcortical white matter	[54,56]
Organic acidurias, GM1 and GM2 gangliosidoses, guanidinoacetate methyltransferase deficiency	May at times present with T2 hyperintense bilaterally symmetric brainstem nuclei or basal ganglia lesions, often in addition to more canonical white matter or other intracranial disease	[26–28]
**Acquired neurologic insult**		
Hypoxic ischemic injury (HII)	MRI may present with T2 hyperintense lesions of the bilateral putamina and thalami ± bilateral globi pallidi lesions and white matter lesions. HII after the neonatal period tends to spare the thalami.	[12,23]
Hypoglycemic ischemic encephalopathy	Classically involves the occipital cortex in addition to basal ganglia, thalami. T2 lesions preceded by diffusion restriction may evolve into encephalomalacia.	[29]
Refractory status epilepticus	Typically involves pulvinar (+/- cortex), accompanied by diffusion restriction, histopathology similar to Wernicke-Korsakoff lesions.	[30]
**Toxic/metabolic**		
Wernicke-Korsakoff Syndrome (thiamine deficiency)	Uniformly involves mammillary bodies in addition to structures affected in LS; basal ganglia involvement is rare in classical (alcohol use disorder- associated) disease but can occur in pediatric and nonalcohol use disorder- associated adult cases; histology differs from LS in that petechial hemorrhage is often observed.	[31]
Kernicterus (CNS bilirubin toxicity)	T2 hyperintense basal ganglia lesions have been reported as late as 1 year; some reports involve T1 hyperintense lesions.	[32]
Osmotic myelinolysis	Most commonly occurs in individuals with comorbid alcohol use disorder, cases with thalamic and striatal T2 signal changes have been reported.	[33]
Carbon monoxide poisoning	Bilateral globi pallidi are uniformly T2 hyperintense; cerebral cortex, cerebral white matter, cerebellum, midbrain may also be involved. In rare cases, T2 and T1 hyperintensity of the globi pallidi have been observed. Lesions may regress with time.	[34]
Metronidazole encephalopathy	Lesions mostly commonly occur in the brainstem and/or cerebellar dentate nuclei, although basal ganglia are rarely involved. Metronidazole encephalopathy is more common in individuals with comorbid alcohol use disorder.	[35]
Fedratinib toxicity	Reported as rare side effect in individuals being treated for myeloproliferative disorders. T2 hyperintense lesions were observed in the bilateral caudate nuclei, lenticular nuclei, and thalami but not in the mammillary bodies. Clinical and radiographic improvements were reported after thiamine supplementation.	[13]
Disulfiram toxicity	Bilateral globi pallidi and putamina are affected. Most occurrences in individuals with alcohol use disorder being treated with disulfiram; however, one case reported in a child after accidental ingestion.	[14]
Carbon disulfide toxicity	This industrial solvent is metabolized by cytochrome P450 enzymes to thiocarbamide, 2-mercapto-2-thiazolinone-5, and 2-thiothiazolidine-4- carboxylic acid, the latter of which conjugates to glutathione. Chronic exposure leads to encephalopathy, parkinsonism, and neuropathy. Imaging features may be suggestive of a microangiopathy.	[15]
Vigabatrin exposure	Lesions occur in ~30% of patients with epilepsy treated with vigabatrin (a GABA transaminase inhibitor) and are diffusion restricting as well as T2 hyperintense. Lesions resolve with discontinuation of therapy.	[16]
**Infection-related**		
Hemolytic-uremic syndrome (HUS)	Lesions often involve splenium of the corpus callosum in addition to structures typically affected in LS, were diffusion-restricting and T2 hyperintense, and resolve in most cases. Edema, necrosis, spongiosis, gliosis, hemorrhages, and thrombotic microangiopathy have all been reported on histology.	[17]
Flavivirus (West Nile, Murray Valley Encephalitis)	Lesions occur in a significant proportion of patients and may be diffusion restricting and T2 hyperintense or diffusion restricting (and resolving) only, with the latter seeming to confer a better prognosis.	[18]
Variant Creutzfeldt-Jakob disease (bovine spongiform encephalopathy)	Pulvinar most commonly affected, dorsomedial thalamic nuclei, caudate head, and periaqueductal gray matter may also be involved. Lesions may regress. Histology is characterized by spongiform change, neuronal loss, astrocytosis, and deposition of partially protease-resistant prion protein.	[19]
Influenza A, B associated encephalopathy/Reye syndrome	Involvement of the splenium of the corpus callosum is often reported in addition to the gray matter structures affected by LS.	[20]
Cerebral malaria	Lesions of gray and white matter have been reported with some authors interpreting the gray matter lesions as resulting from vasogenic edema.	[21]
**Other genetic disorders**		
Wilson disease (ATP7B)	The MRI brain may be normal, but the most common abnormality is a T2 hyperintense signal in the lateral rim of the bilateral putamina. T2 hyperintensity of the caudate, globi pallidi, and thalami were seen only when putaminal lesions were absent. Rarely, T1 hyperintensity may be seen in the thalami. Response of brain lesions to copper chelation remains unclear.	[22]
Menkes disease (ATP7A)	T2 hyperintense lesions of the caudate nuclei, lenticular nuclei, and globi pallidi have been reported. Isolated T2 hyperintensities of the parieto-occipital white matter and isolated cerebral atrophy have also been reported.	[24]
Juvenile Huntington disease *(HTT)*	Caudate atrophy, similar to adult patients, was observed in addition to T2 signal abnormalities of the caudate, which is not a feature of adult-onset disease. Interestingly, juvenile onset HD mostly typically presents with Parkinsonism, although adult-onset disease is characterized by chorea.	[25]
Dentatorubral-pallidoluysian atrophy *(ATN1)*	T2 hyperintensity of the globi pallidi and thalami are present with atrophy of the tegmentum, other deep gray nuclei, and cerebellum. Histopathology demonstrates characteristic neuronal intranuclear inclusions.	[26]

MRI, magnetic resonance imaging; OXPHOS, oxidative phosphorylation; GABA, Gamma-Aminobutyric Acid; HD, Huntington Disease; CNS, central nervous system.

**Table 2:** Published cases of Leigh syndrome reporting radiographic improvement.

Gene or biochemical defect	Age initial imaging	Age repeat imaging	Reversing lesion(s)	References
n/r	10mo	17mo	Basal ganglia	[7]
n/r	10mo	17mo	Basal ganglia	[7]
n/r	22mo	30mo	Right caudate, midbrain	[7]
n/r	10yr 11mo	11yr 7mo	Left caudate	[8]
n/r	3yr	3.5yr	Basal ganglia	[43]
PDHC deficiency	1yr	2yr	Globus pallidus, cerebellum	[44]
Complex I deficiency	n/r	n/r	Basal ganglia	[42]
PDHC deficiency	4yr 7mo	4yr 9mo	Basal ganglia	[42]
n/r	n/r	n/r	Upper brainstem	[39]
Partial COX deficiency	22yr	22yr	Basal ganglia, thalami	[45]
n/r	14mo	27mo	Globi pallidi	[46]
*PDHA1*	3yr	7yr	Basal ganglia, inferior olivary nuclei	[48]
*PDHA1*	n/r	n/r	Basal ganglia, cerebellum	[41]
*MT - ND5*	n/r	n/r	Midbrain	[41]
*MT-ATP6*	n/r	n/r	Basal ganglia	[41]
*HIBCH* (c.287C > A)	14mo	24mo	Basal ganglia	[47]
*MT-ND3* (m.10197G > A)	16yr	17yr	Thalamus, cerebral peduncle, pons, medulla oblongata	[49]
*PDHA1*	1yr 8mo	6yr 8mo (multiple)	Basal ganglia (initially improved, then progressed)	[40]
*SLC19A3*	2.5yr	3yr	Basal ganglia	[54]
*MT-ATP6* (m.8689G > A)	n/r	n/r	Putamen	[9]
*MT-ND3* (m.10158T > C)	n/r	n/r	regression reported, lesion location not detailed	[9]
*MT-ATP6 (m.9176 T> C)*	n/r	n/r	regression reported, lesion location not detailed	[9]
*TPK1*	21mo	29mo, 3yr 2mo, 3yr 5mo, 6yr 1mo	Basal ganglia, dentate nuclei	[56]
*SUOX* (c.1096C > T; c.1376G > A)	1yr	2yr, 6.5yr	Globi pallidi, substantia nigra	[53]
*SUOX* (c.1096C > T; c.1376G > A	1yr 2mo	2yr 8mo	Globi pallidi, substantia nigra	[53]
*SUOX* (c.1096C > T; c.1376G > A)	1yr 4mo	3yr 10mo	Globi pallidi, substantia nigra	[53]
*NDUFS4* (c.355G > C)	1yr	2yr 5mo 3yr 9mo	Thalami, brainstem (f/u MRI showing new abnormal signal in medulla)	[52]
*MT-ATP6* (m.8993T > G)	n/r	32mo later	Putamen	[50]
*MT-ND4* (m.11778G > A)	n/r	n/r	Brainstem and basal ganglia lesions at	[50]
*MT - ND6* (m.14484T > C)			baseline; only oculomotor nuclei in the f/u study	
*MECR*	n/r	n/r	Brainstem and basal ganglia at baseline; mildly evident basal ganglia in the f/u study	[50]
*PDHA1* (c.1132C > T)	16mo	28mo	Globi pallidi	[38]
*PDHA1* (c.615C > G)	1yr 9mo	4yr 1mo	Globi pallidi	[38]
*NDUFAF3*	9mo	5yr	Caudate, putamen, thalami, red nuclei	*Case 1*
*PDHA1*	16mo	4yr	Dentate nuclei	*Case 2*

n/r, not reported; f/u, follow up; PDHC, pyruvate dehydrogenase complex; COX, cytochrome C oxidase; MRI, magnetic resonance imaging.
